# Clinical and Educational Aspects of Neuroimaging in Microsurgery of the Posterior Fossa: A Comprehensive Review

**DOI:** 10.7759/cureus.60730

**Published:** 2024-05-21

**Authors:** Levent Tanrikulu

**Affiliations:** 1 Neuro-Oncology, University of Marburg, Marburg, DEU

**Keywords:** microsurgery, education, virtual reality, neurovascular conflicts, neuroimaging

## Abstract

The clinical and educational value of modern high-resolution magnetic resonance imaging (MRI) and image processing in neurovascular diseases of the posterior fossa with regard to preoperative planning and intraoperative comparison with the actual anatomical situation was consecutively evaluated. Patients with trigeminal neuralgia (TN), hemifacial spasm (HFS), glossopharyngeal neuralgia (GN) and arterial hypertension (HTN) were analyzed. The high-resolution MRI data was segmented and visualized three-dimensionally using computer graphics methods. New anatomical insights were gained, such as the classification of neurovascular compression (NVC) in HFS and GN. It was also possible to visualize the pathognomonic cerebrospinal fluid signal in patients with TN for the first time. Using the new imaging methods, pregnant hypertensive patients were examined and the existence of NVC was confirmed for the first time, and the findings were compared to other studies dealing with NVC syndromes. This review gives an overview on the established methods of neuroimaging and image processing of neurovascular structures in the posterior fossa with the focus on clinical and educational aspects.

## Introduction and background

Neurovascular compression (NVC) refers to a noticeable, pathological contact or compression between cranial nerves and vessels in the posterior fossa [1]. This compression can occur along the entire cisternal course of a cranial nerve, whereby the so-called root entry zone (REZ) of a cranial nerve at the transition from the central to the peripheral myelin of the nerve fibers is a predilection site for NVC [2-5]. This region varies in length for the different cranial nerves and is located close to the brainstem. Trigeminal neuralgia (TN), hemifacial spasm (HFS) and glossopharyngeal neuralgia (GN) are typical disease entities in which NVC is the cause in the majority of cases [2,3,6]. There is also evidence that a number of other diseases, such as essential arterial hypertension (HTN) and certain forms of dizziness and tinnitus, are also associated with NVC [7-11].

The therapeutic options are divided into causal and destructive treatment modalities. Today, microvascular decompression (MVD) as described by Jannetta et al., is the recognized causal method of choice for the successful treatment of such syndromes [11,12].

## Review

General remarks

Microvascular decompression (MVD) is a surgical procedure with the aim of eliminating the compression of an affected nerve by a vessel. A circumscribed craniotomy or craniectomy (approx. 3.5 cm) behind the ear is used to access the nerves in the posterior fossa. The affected cranial nerve is approached under micro-neurosurgical conditions. Neuro-navigation can be applied to a more secure estimation of the course of the transverse and sigmoid sinuses, because the asterion not always represents a reliable landmark of their junction. The symptomatic compression site between the vascular loop and the cranial nerve is exposed micro-surgically. The blood vessel, typically an artery, is carefully separated from the nerve and repositioned without compromising the vessel's perfusion. The interposition of various materials (teflon, dacron, piece of patty, muscle) between the vessel and the cranial nerve prevents the vascular loop from falling back into its original position [10,11].

This makes it possible to successfully and permanently treat the corresponding symptoms in a large number of cases. The risk of functional impairment to the cranial nerve is lower compared to destructive procedures like rhizotomy. In addition to the general risks of surgery, the specific risk of MVD is hearing impairment or hearing loss; the risk of hearing loss is approximately 1% with trigeminal neuralgia (TN), approximately 6-10% with hemifacial spasm (HFS) and less than 1% with glossopharyngeal neuralgia (GN) [11]. In order to make the procedure safe for the patient and to avoid functional limitations, all phases of the surgery are monitored with the support of modern intraoperative neuro-physiological monitoring. This involves monitoring of the brainstem evoked response-audiometry and the electromyographic activities of the corresponding cranial nerves (trigeminal nerve, facial nerve, glossopharyngeal nerve), and integrating them into the surgical process [11,12].

Trigeminal neuralgia

Trigeminal neuralgia (TN) is a facial pain in the area supplied by the fifth cranial nerve (trigeminal nerve, CN V). The pain can occur in one or more branches of the nerve and is classically described as electrifying and shooting. In 5% of the cases, the pain occurs in both sides of the face, but the condition is usually unilateral. The pain occurs in attacks, lasting seconds to minutes. The pain can be triggered by eating, laughing, brushing teeth, talking, shaving or simply touching the face. In rare cases, a tumor in the course of the nerve or a focus of demyelination in the core area of the nerve, as part of multiple sclerosis, can be identified as the cause of TN. These causes of secondary TN can be ruled out using MRI. In the majority of idiopathic TN, an abnormal vascular course can be identified, usually by the superior cerebellar artery (SCA), less frequently by the anterior inferior cerebellar artery (AICA), which leads to compression of CN V in the area of the REZ.

If TN is secondary to a tumor, surgical removal of the tumor should be considered. Idiopathic TN is initially treated with carbamazepine, phenytoin, baclofen and gabapentin. In cases of resistance to therapy, progression of pain or severe side effects of medication, surgical procedures are available (causal: MVD, lesional: thermocoagulation, retroganglionic glycerol injection, stereotactic radiosurgical irradiation e.g. using gamma knife). MVD is a causal form of therapy with the aim of surgically correcting the frequently found NVC. Compared to lesional methods, MVD achieves better long-term results in terms of freedom from pain, and preservation of function and quality of life [6,8,10,13,14-16].

Hemifacial spasm

Hemifacial spasm (HFS) is characterized by involuntary contractions (synkinesia) of the facial muscles on one side of the face, particularly around the corner of the mouth and the eye. The painless contractions can appear as individual twitches or as prolonged tonic spasms with contortions of one side of the face. The condition is particularly distressing for those affected and often leads to social withdrawal. Occasionally, the cause can be tumors in the course of the facial nerve, inflammatory changes in the nerve, injuries to the nerve, but also pathological changes in the brainstem. In most cases, however, there is NVC of the nerve at the facial REZ. Carbamazepine, phenytoin and baclofen are used as medication. Symptomatically, botulinum toxin is injected into the affected facial muscles, which leads to paralysis of the corresponding muscles for an average of three months [17]. MVD should be used as the causal therapy. The procedure can lead to lasting success without functional impairment in around 80-90% of cases [7,18-20].

Glossopharyngeal neuralgia

Glossopharyngeal neuralgia (GN) is a pain in the area of the throat and base of the tongue and can also affect the external auditory canal in extreme phases. The pain is described as sharp, shooting, lancinating and electrifying. It can sometimes have a "devastating" character. The pain can occur suddenly and spontaneously, but can also be triggered by swallowing, speaking, coughing or touching the tongue or throat. GN can be accompanied by cardiac arrhythmia and high blood pressure. In rare cases, the cause can be tumors in the course of the nerve or the neck, inflammatory changes in the nervous system or injuries to the nerve. In the majority of cases, no definite cause is found, so that GN is referred to as idiopathic GN. In most cases, a conspicuous vascular loop is found at the REZ of the glossopharyngeal nerve and vagus nerve close to the brainstem. Treatment is mainly with carbamazepine and phenytoin. In addition, various methods involving the infiltration of anesthetics or alcohol are used at various extracranial sites along the course of the nerve with predominantly temporary pain relief. MVD is successful in achieving permanent pain relief without functional impairment, so that this treatment method is increasingly regarded as the method of choice [11].

Neurovascular hypertension

Over 80% of patients with high blood pressure suffer from so-called essential hypertension, for which no clear cause has yet been found. If left untreated, this disease often leads to arteriosclerosis, strokes and heart attacks. Many patients therefore have to take medication for the rest of their lives. It is known that the central nervous system plays a major role in regulating blood pressure. In some patients with essential hypertension, noticable compressions of the vertebral artery or the posterior inferior cerebellar artery (PICA) with the rostral ventrolateral medulla oblongata (RVLM) in the sense of an NVC have been observed [9]; in these cases, the term neurovascular hypertension (nHTN) is also used. In a selected number of patients, it has been shown that MVD can reduce or even completely normalize blood pressure [9].

Imaging and image processing of NVC syndromes

Today, it is possible to visualize NVC in a large number of patients with the above-mentioned complaints with special high-resolution MRI and anatomical experience. The relationships between the cranial nerves and the vessels on the surface of the brainstem are characterized by a complex, three-dimensional (3D) topography. The spatial understanding of these relationships is necessary for the surgical treatment of NVC syndromes, as well as space-occupying processes in the cerebellopontine angle.

Up to now, MRI data sets without high resolution have usually been used. The imaging was essentially limited to a two-dimensional (2D) presentation of these structures [21-24]. This significantly limited the assessment of the 3D cisternal course of the individual vessels and cranial nerves. As a result, existing vascular-nerve conflicts were often overlooked. In cases of recognizable conflicts, it was hardly possible to differentiate between arterial and venous compression unless the neuroradiologist/neurosurgeon had extensive experience.

**Figure 1:**
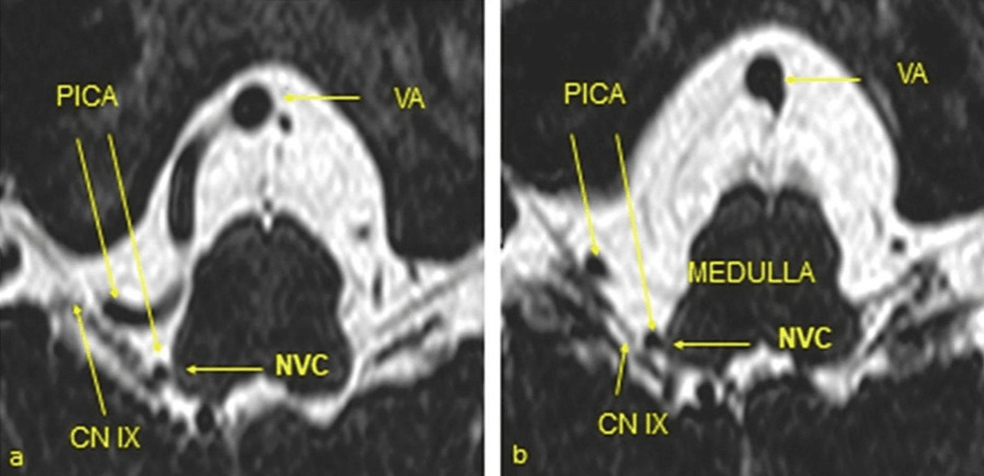
High-resolution, heavily T2-weighted MRI-CISS sequence with right-sided neurovascular compression (NVC) in right-sided glossopharyngeal neuralgia (a and b). Right-sided vascular loop of the posterior inferior cerebellar artery (PICA) originating from the vertebral artery (VA) with formation of an NVC at the root entry zone of the glossopharyngeal nerve (CN IX) at the ventrolateral medulla oblongata (CN: cranial nerve). Taken from Tanrikulu et al. with permission of the main author (published under Creative Commons license) [25].

3D representations of the neurovascular relationships on the surface of the brainstem can now be robustly generated from the underlying multimodal image data. Using image morphological filtering and volume growing, the anatomical neurovascular structures are extracted and segmented (see Figure 1).

**Figure 2:**
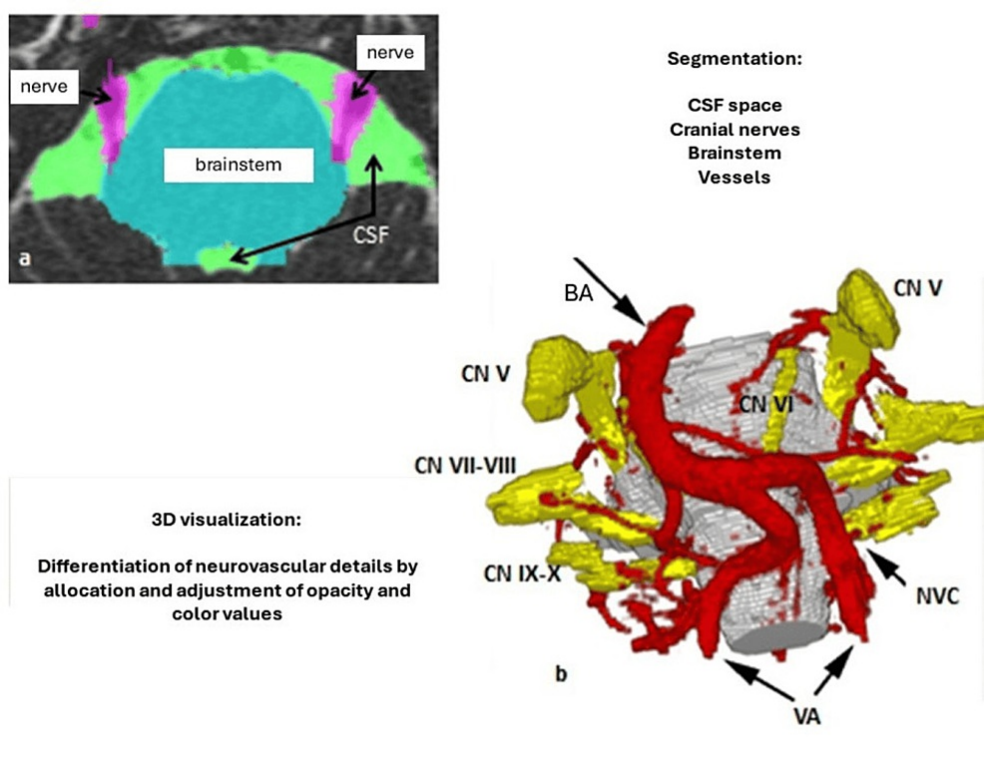
Segmentation and 3D visualization. a) Segmentation is performed in three steps: anisotropic diffusion and morphological filtering ((generation of a uniformly highly hyperintense cerebrospinal fluid space, (CSF space), green color coding)) and extraction of the CSF space including the defined nerves (pink color coding) and the brainstem (cyan color coding) by means of volume growing. Each voxel is assigned a color and an opacity so that the cerebrospinal fluid space appears transparent in the 3D visualization (see 2b) and the segmented vessels and the brainstem appear opaque. Taken from Tanrikulu et al. with permission of the main author (published under Creative Commons license) [25].

Based on a non-linear registration between an anatomical atlas and topographic MRI volume data, a 3D visualization is produced that contains the individual neurovascular structures. The optimal information of relevant structures is extracted from the different imaging sequences, resulting in an optimized 3D representation after registration (see Figure 2).

High-resolution, heavily T2-weighted CISS (constructive interference in steady state) sequences serve as the basis for all procedures. The developed methods are used in preoperative surgical planning and intraoperatively during MVD.

**Figure 3:**
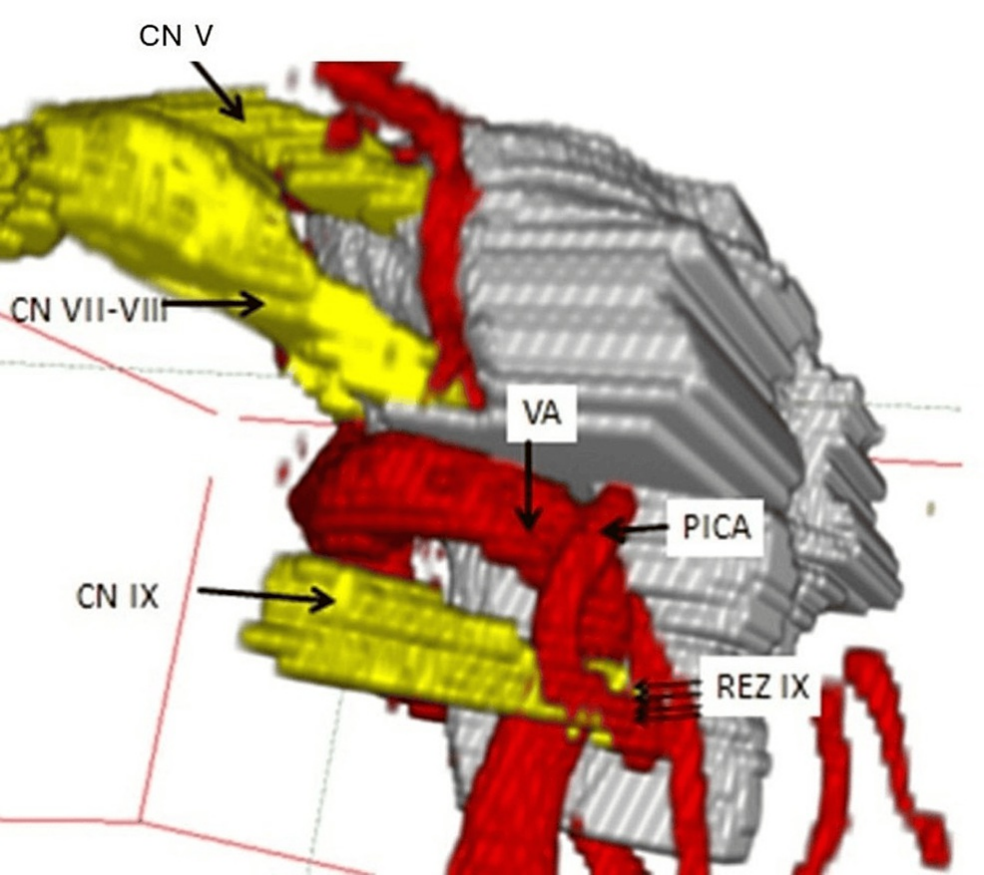
Lateral view of the 3D visualization of left-sided glossopharyngeal neuralgia. The neurovascular compression at the REZ of the glossopharyngeal nerve (CN IX) is caused by a so-called "sandwich" compression on the part of the vertebral artery (VA) and the posterior inferior cerebellar artery (PICA). CN V: trigeminal nerve, CN VII-VIII: facial-vestibulocochlear nerve complex, REZ: Root entry zone. Taken from Tanrikulu et al. with permission of the main author (published under Creative Commons license) [25].

By means of morphological filtering (extraction of the green defined cisternal subarachnoid space including the essential vascular structures, the pink defined cranial nerves and the cyan marked brain stem) and computer graphic non-linear registration, the entire cisternal volume is visualized into a three-dimensional, dynamic object using a volume growing function (see Figures 2 and 3).

The dynamic 3D visualization can be moved and zoomed interactively from all spatial planes on the monitor (see Figure 3). This means that the surgical site to be encountered intraoperatively can already be anticipated preoperatively based on the real patient anatomy.

With high-resolution MRI the essential morphological parameters in patients with classic TN was analyzed [26]. 180 patients with TN received a high-resolution MRI-CISS and TOF (time of flight, MR angiography) sequence.

The following morphologic parameters were investigated: relation between the NVC site (caudal/cranial/laterocaudal/mediocranial) and the facial pain area (V1, V2, V3), nerve deformity, compressing vascular loop, existence of CSF signal caused by differentiation or pushing apart of trigeminal fascicles by a vascular loop, and localization of the compressing vascular loop. Ten patients with V1 affection had six caudal, four mediocranial, but no cranial and no laterocaudal NVCs. 26 patients with V2 affection showed 17 caudal, no cranial, one laterocaudal and eight mediocranial NVCs.

29 patients with V3 affection showed 23 caudal, one cranial, three laterocaudal and two mediocranial NVCs. 25 patients with combined V1-V2 affection showed 17 caudal, one cranial, no laterocaudal and seven mediocranial NVCs. 36 patients with combined V2-V3 affection showed 30 caudal, three cranial, one laterocaudal and two mediocranial NVCs. Six patients with a combined V1-V2-V3 affection showed four caudal, one cranial, no laterocaudal and one mediocranial NVC. 63 patients (35%) showed a deformity of CN V due to distortion of the trigeminal fascicles by the compressive vascular loop. 37 of 39 patients (95%) with right-sided deformity showed right-sided TN. 21 of 22 patients (95%) with left-sided TN showed left-sided deformities. Two patients with bilateral nerve deformity showed bilateral TN [26].

Rostral NVC through the superior cerebellar artery (SCA) was seen in 24 patients (17%). Caudal NVC by the SCA was seen in 10 patients (7%). Double arterial NVC by the SCA was seen in 33 cases (23%). A so-called "sandwich" compression was seen in 33 patients (23%). A CSF signal was seen in 24 patients. All 24 patients had a V1 affection. The most frequent compressive loops ran from the cranial direction to the REZ of the CN V. In this study, it was possible to categorize the examined patients into rostral vascular loop, caudal vascular loop, venous compression and so-called "sandwich" compression. In particular, it could be shown that the observed CSF signal was pathognomonic for a V1 affection and did not occur on the asymptomatic side [26].

In another study an anatomical analysis of the facial and vestibulocochlear nerves and a representation of the variable vascular relationships by using the 3D visualization in patients with HFS was investigated [27]. 25 patients (m/f: 13/12) with HFS were examined using high-resolution MRI-CISS sequence. This was followed by 3D visualization using segmentation and direct volume visualization. The segmentation and 3D visualization of the facial-vestibulocochlear complex were taken into account with special consideration of the REZ of these cranial nerves and their proximal (REZ to the nerve crossing / decussatio of the facial/ vestibulocochlear nerve) and distal nerve segment (decussatio of the nerves to occur in the internal auditory canal) including the accompanying blood vessel loops. MVD was carried out in 20 out of 25 patients (80%). In these cases, 3D visualization was compared to the microsurgical findings. The neurovascular anatomy was also analyzed and compared on the symptomatic and asymptomatic side based on 3D visualization. On the symptomatic sides, the AICA was the most common compressive vascular loop. The PICA, VA, the internal labyrinthine artery and venous vessels were also found as causal vessels for the NVC. In addition, three distinct forms of the NVC of the REZ of the facial nerve were shown. On the asymptomatic sides, NVC were also found, especially in the proximal and distal nerve segments of the facial nerve, whereby no NVC was found in the area of the facial nerve. It was shown that the AICA was the most common vascular loop that compressed the proximal and the distal segment of the facial nerve on the asymptomatic sides. The 3D visualization using the high-resolution MRI-CISS data enabled detailed, non-invasive exploration of the facial vestibulocochlear complex and proved to be very advantageous in microsurgical therapy planning [27].

Neurovascular hypertension

Schobel et al. examined the sympathetic activity in pregnant patients with a preeclampsia [28]. This study encouraged us to investigate an association between NVC, HTN and pregnancy. For the first time, NVCs on the rostral, ventrolateral medulla oblongata were detected in pregnant patients with a gestation-induced HTN [29]. A clear limitation of this study was the low number of patients, whereby this can be explained to an MRI examination by the self-evidence of pregnant patients. NVC in the area of the RVLM can result from a pregnancy-related increase in the intravasal blood volume and the increase in cerebral perfusion pressure in the form of a distortion of arterial vessels. This compression may increase sympathetic nerve activity and can therefore cause HTN.

The relevance of NVC in arterial HTN on the RVLM in a more topical study was examined by Sindou et al. [30]. In a series of 201 patients with an HFS, a subgroup of 48 patients with an additional essential arterial HTN with NVC on the RVLM was identified in MRI. In this study, an MVD took place both at the REZ of the facial nerve and the RVLM. 28 patients (58%) were found to be normotone postoperatively and were able to drop their antihypertensive medications with medical care [30].

The NVC in patients with essential arterial HTN will continue to be a very interesting focus for further studies. In particular, there are still no criteria for optimal patient selection to confirm an operation indication in the presence of arterial HTN and NVC. Due to technological advances in imaging, more recent methods will be implemented in an interdisciplinary internal-neurosurgical workflow. In particular, the use of innovative and modern “real-time” MRI representations of an NVC in connection with the measurement of the activity of the sympathetic nervous system and blood pressure as well as to quantify the degree of an NVC on the brainstem appears of high relevance for future projects.

## Conclusions

3D visualization with segmentation and direct volume rendering offers the excellent opportunity to generate new approaches for the virtual, augmented reality of the cranial nerve topography. The anatomical landmarks such as the root entry/exit zones and the cisternal segments are visualized in 3D fashion. This method supports non invasive anatomical and microneurosurgical evaluation of the cranial nerves in the posterior fossa, clinical assessment and sustainable microsurgical training.
